# Hemodynamic and cardiorespiratory responses to submaximal and maximal exercise in adults with Down syndrome

**DOI:** 10.3389/fphys.2022.905795

**Published:** 2022-08-19

**Authors:** Guillermo R. Oviedo, María Carbó-Carreté, Myriam Guerra-Balic, Nauris Tamulevicius, Laura Esquius, Joan Guàrdia-Olmos, Casimiro Javierre

**Affiliations:** ^1^ Faculty of Psychology, Education and Sport Science Blanquerna, University Ramon Llull, Barcelona, Spain; ^2^ School of Health Science Blanquerna, University Ramon Llull, Barcelona, Spain; ^3^ Serra Hunter Fellow, Department of Cognition, Development and Educational Psychology, Faculty of Psychology, University of Barcelona, Barcelona, Spain; ^4^ Institute of Neuroscience, University of Barcelona, Barcelona, Spain; ^5^ Department of Health Sciences and Human Performance, College of Natural and Health Sciences, The University of Tampa, Tampa, FL, United States; ^6^ Foodlab Research Group, Faculty of Health Sciences, Universitat Oberta de Catalunya, Barcelona, Spain; ^7^ Department of Social Psychology and Quantitative Psychology, Faculty of Psychology, University of Barcelona, Barcelona, Spain; ^8^ Universitat de Barcelona Institute of Complex Systems, Barcelona, Spain; ^9^ Department of Physiological Sciences, Faculty of Medicine, University of Barcelona, Barcelona, Spain

**Keywords:** down syndrome, cardiorespirarory fitness, blood preasure, exercice, hemodymamics

## Abstract

**Introduction:** The genetic disorder causing Down syndrome (DS) affects the cardiorespiratory and hemodynamic parameters. When exercising, sufficient blood flow is necessary for active muscles. Cardiac output (Q) must be proportional to the peripheral requirements. In case the stroke volume (SV) is lower, the heart rate (HR) will increase further in order to maintain an adequate blood flow in the active territories (HR compensatory response). People with DS have a lower HR response to maximal exercise. Nevertheless, the response of the hemodynamic and cardiorespiratory parameters during the submaximal phases of maximal exercise was not well studied.

**Objective:** to evaluate cardiorespiratory and hemodynamic parameters 1) during submaximal and 2) maximal metabolic treadmill test in individuals with and without DS.

**Methods:** fifteen adults with DS (age = 27.33 ± 4.98 years old; n = 12 males/3 females) and 15 adults without disabilities, matched by age and sex, participated in this cross-sectional study. Peak and submaximal cardiorespiratory and hemodynamic parameters were measured during a treadmill test. Linear mixed-effects models were used to analyse interactions between the variables. Post-hoc analyses were employed to assess within and between-group differences.

**Results:** The DS group showed lower peak values for ventilation (VE), respiratory exchange ratio (RER), tidal volume (V_T_), ventilatory equivalent for O_2_ (VEqO_2_), end-tidal partial pressure for O_2_ (P_ET_O_2_), O_2_ uptake (VO_2_) and CO_2_ production (all *p* < 0 .050), Q, SV, systolic and diastolic blood pressure (SBP, DBP), and HR (all *p* < 0 .050). There were group-by-time interactions (all *p* < 0 .050) for all ventilatory submaximal values. Significant group and time differences were observed for VE; RER; respiratory rate (RR); VEqO_2_; P_ET_O_2_; VO_2,_ and V_T_ (all *p* < 0 .050). There were also group-by-time interactions (all *p* < 0 .050) and group and time differences for SBP, mean arterial blood pressure (MAP) and HR (all *p* < 0.010).

**Conclusion:** During submaximal exercise, we verified a compensatory response of HR, and greater VE and VO_2_ in the individuals with DS. In addition, we were able to observe that the DS group had a reduced SBP and MAP response to submaximal exercise. On the other hand, we found that adults with DS have lower peak hemodynamic and cardiorespiratory values, and a lower cardiac reserve. Further research is warranted to investigate the effects of these results on the general health of adults with DS and the impact of long-term exercise programs on these parameters.

## 1 Introduction

Down syndrome (DS) is a chromosomal disorder that occurs in the human species, which has a variable and a wide range of impact and severity at an individual level. This genetic disorder influences physical and clinical characteristics, cardiorespiratory fitness, and intellectual disability (World Health Organization, 2000). European DS prevalence during 2011–2015 was estimated at 4.9 per 10,000 inhabitants, which corresponds to approximately 359,000 people with DS living in Europe, of whom 35% are under the age of 20 and 35% above 40 years old (De Graaf et al., 2018).

Down syndrome individuals’ life expectancy and survival have increased significantly since the 1960s, with median life expectancy in the late 50 s (Englund et al., 2013; Glasson et al., 2016). This is secondary to better preventive health care, educational programs, curative surgical intervention on congenital and gastro-intestinal anomalies, and social support. Some predictors of life expectancy include race, sex, birth weight, gestational age at birth, and presence of heart defects and other structural anomalies (Yang et al., 2002; Englund et al., 2013). Infectious diseases, mainly pneumonia, followed by congenital heart defects, circulatory disease, and dementia, are the leading causes of death in individuals with DS (Englund et al., 2013). Also, one of the most significant studies analysing cardiovascular abnormalities in DS identified that 342 of 821 (42%) DS infants born from 1985 to 2006 had cardiovascular anomalies (Irving and Chaudhari, 2012).

Individuals with DS have a broad spectrum of cardiovascular disease, and multiple studies have found that their response to exercise is different from non-DS individuals (Mendonca et al., 2010; Fernhall et al., 2013). Baynard et al. (2008) reported that the peak oxygen consumption (VO_2_peak) in adolescents and adults with DS was similar to 60-year-old non-disabled adults. Moreover, other studies have proposed that individuals with DS would have autonomic dysfunctions such as decreased responses to sympathetic nervous system stressors, altered baro-reflex sensitivity, cardiovascular chronotropic incompetence, and altered hormonal response to exercise (Heffernan et al., 2005; Bricout et al., 2008; Fernhall et al., 2013; Hilgenkamp et al., 2019). Interestingly, it was found that at rest, individuals with DS have higher parasympathetic activity than their peers without disabilities, but during the exercise these differences disappeared. Therefore, the authors suggest that low-intensity exercise may facilitate an adequate increase in heart rate (HR), and other variables may be responsible for the inability to increase HR as expected during maximal exercise (Baynard et al., 2004).

It is documented that at rest and during one set of light strength exercise, parameters such as cardiac output (Q), stroke volume (SV), and HR are lower in people with DS (Vis et al., 2012a). In addition, previous work shows that there are higher metabolic demands in people with DS versus non-DS at submaximal intensities of exercise, which makes DS persons less efficient when performing physical activities or exercise (Agiovlasitis et al., 2009).

We think that it is important not only to determine these parameters during maximal exercise, as other researchers have done, but also during submaximal exercise. As part of daily life, people perform physical activity at submaximal intensities. As Oppewal et al. (2020) indicate, even the performance of exercises whose energy expenditure corresponds to low intensities will benefit the health of persons with intellectual disabilities. Therefore, investigating the response of hemodynamic and cardiorespiratory parameters, not only during maximal exercise but also at submaximal intensities, can provide insight into the response and function of these parameters at magnitudes that are more representative of activities of daily living.

Thus, the main objectives of our study were to analyse and compare the responses of cardiorespiratory and hemodynamic parameters during maximal and submaximal exercise in individuals with and without DS matched by age and sex.

## 2 Methods

### 2.1 Study design and participants

This cross-sectional study included three women and 12 men with DS (27 ± 5 years old) and 15 adults without disabilities (non-DS) with similar ages and sex. Participants with DS from three occupational day centers were invited for this study. Adults without DS were recruited from Universities’ campuses. A previous study where cardiorespiratory parameters were assessed in adults with and without DS, showed a large effect size (Hilgenkamp et al., 2018). Therefore, after calculating the sample size (power = 0.80; *α* < 0.05; effect size of *d* = 1), we determined that we needed to recruit at least 28 participants (14 in each group).

Interested persons between 18 and 35 years old with and without DS, matched by age and sex, were invited to the laboratory facilities. Before obtaining the informed consent of participants and parents/legal guardians, we explained the study protocol, benefits and risks. Next, all participants were given ample time to read the study protocol and ask all necessary questions. Finally, all volunteers undergo a physical examination to disclose any physical and/or cardiovascular pathology that would make maximal exercise contraindicated.

To be part of the present study, all participants needed to be able to walk without aids; be willing to perform a treadmill test; and parents/legal guardians and participants should have signed the informed consent. In addition, exclusion criteria were: taking medications that could affect physical performance or HR; having any cardiovascular disease or other contraindications to exercise.

This study was approved by the Institutional Review Board (CER URL 2017_2018_008) and complied with the principles of the Declaration of Helsinki (World Medical Association, 2013).

### 2.2 Testing procedure

We organized one to three familiarization sessions so that participants could become acquainted with the tests and equipment used in this study. All tests were performed during the morning, and all participants were requested to neither take part in moderate or vigorous exercise nor consume alcohol and/or caffeine for at least 24 h before the testing day.

#### 2.2.1 Anthropometric measurements

We measured participants’ height (Seca 225, Seca, Hamburg, Germany) and weight (Tanita MC-780U, Arlington Heights, IL, United States) to the nearest 0.1 cm and 0.1 kg, respectively. Finally, we calculated the body mass index (BMI) for every participant by using the equation weight (kg)/height (m^2^).

#### 2.2.2 Cardiorespiratory fitness assessment

Participants walked on a treadmill (Quasar model, HP Cosmos sports and medical gmbh, Nussdorf-Traunstein, Germany) at a constant speed (4 km/h), and the gradient increased 2.5% every 2 min until a grade of 12.5% was attained. From this point, grade remained constant, whereas speed was increased 1.6 km/h every minute up to exhaustion. This protocol was used by different authors to assess the cardiorespiratory fitness in persons with DS (Fernhall and Tymeson, 1987; Mendonca and Pereira, 2009; Boer and Moss, 2016; Hilgenkamp et al., 2018). Peak values were calculated from the average of the last 30 s of exercise. Peak effort was identified by a respiratory exchange ratio (RER) > 1.0, or HR and/or VO_2_ plateau and when a participant could no longer continue. Values were recorded continuously, and every minute’s average was used for the submaximal analysis. In this case, the highest submaximal workload was considered as the one exceeded by 90% of our participants.

We measured the respiratory gas exchange with an automatic gas analysis system (Metasys TR-plus, Brainware SA, La Valette, France) and using a two-way mask (Hans Rudolph, Kansas, United States). Before each test, we performed gas and volume calibrations. In addition, we used a 12-lead electrocardiogram to monitor the HR of the participants (CardioScan v.4.0, DM Software, Stateline, Nevada, United States).

#### 2.2.3 Hemodynamic assessments

As in previous studies performed in our laboratory (Esquius et al., 2019; Oviedo et al., 2021), we used a finger cuff to obtain beat-to-beat hemodynamic and blood pressure (BP) information (Nexfin, BMEYE Amsterdam, Netherlands). Stroke volume and Q are derived by pulse contour method using the measured systolic pressure-time integral and the afterload of the heart (Wesseling et al., 1993). The finger cuff was placed around the middle phalanx of the left middle finger, and the arm was placed on a platform and secured with elastic straps to prevent any movement. We monitored the finger photoplethysmography continuously, and values were averaged every minute. Peak values were obtained from the last 30 s of the treadmill test. For the present analysis, all data obtained in each time point were visually inspected and values containing a variation higher or lower than two SD were eliminated.

### 2.3 Statistical analysis

Descriptive statistics were calculated for all variables. We used the Kolmogorov-Smirnov and Shapiro Wilk tests to check the normality of the data.

The interactions between group (DS vs. non-DS) and condition (different workloads) were analysed using a linear mixed-effects model. In addition, post-hoc comparisons with Bonferroni correction were conducted to analyse within and between-group differences. Finally, to examine between-group differences in characteristics, cardiorespiratory and hemodynamic peak values, independent *t*-tests were conducted, and effect size (Cohen’s *d*) was calculated when possible with 0.2; 0.5 and 0.8 indicating a small, medium and large effect, respectively (Cohen, 1988).

Statistical analyses were performed with the Statistical Package for the Social Sciences version 25.0 (IBM SPSS, Chicago, IL, United States). Statistical significance was set at an alpha level < 0.050 (*p* < 0.050).

## 3 Results

The general characteristics of the participants are presented in Table 1. Both groups had similar weights. However, individuals with DS were shorter and had a higher BMI than the non-DS participants (all *p* < 0.050). Regarding cardiorespiratory and hemodynamic parameters at rest, we did not observe between-groups significant differences (Table 2).

**TABLE 1:** Participants’ characteristics.

	Non-DS (n = 15)		DS (n = 15)				*p*-value	Effect size
Characteristics								
Sex (male/female)	12/3			12/3			1.000	--
Age (years)	27.3	±	5.0	27.3	±	5.0	1.000	--
Weight (kg)	71.5	±	9.9	66.6	±	9.9	0.185	0.50
Height (m)	1.75	±	0.09	1.56	±	0.06	<0.001	2.40
BMI (kg/m^2^)	23.3	±	1.6	27.4	±	4.3	0.002	1.28

values are mean ± standard deviation.

Abbreviations; DS (Down syndrome); BMI (body mass index).

**TABLE 2:** Participants’ cardiorespiratory and hemodynamic values at rest.

	Non-DS (n = 15)			DS (n = 15)			*p*-value	Effect size
	Resting values			Resting values				
**Cardiorespiratory data**								
VE (L•min^−1^)	10.1	±	1.7	9.9	±	2.9	0.720	0.13
RER	0.88	±	0.04	0.85	±	0.05	0.416	0.30
RR (breath•min^−1^)	18.3	±	4.1	20.8	±	6.4	0.162	0.52
V_T_ (L)	0.50	±	0.13	0.48	±	0.11	0.171	0.51
VEqO_2_	31.1	±	4.8	29.2	±	4.4	0.273	0.41
VEqCO_2_	108.2	±	6.1	106.7	±	4.2	0.139	0.56
P_ET_O_2_ (mmHg)	37.8	±	5.7	34.8	±	4.9	0.581	0.20
VO_2_ (ml•kg^−1^•min^−1^)	4.5	±	0.9	5.0	±	1.1	0.208	0.47
VO_2_ (L•min^−1^)	0.32	±	0.06	0.33	±	0.11	0.423	0.30
VCO_2_ (L•min^−1^)	0.28	±	0.05	0.28	±	0.15	0.368	0.33
**Hemodynamic data**								
Q (L•min^−1^)	6.4	±	2.8	6.5	±	2.6	0.913	0.04
Stroke Volume (ml)	84.3	±	22.7	75.0	±	21.1	0.252	0.42
SBP (mmHg)	137.0	±	26.6	119.5	±	24.8	0.120	0.67
DBP (mmHg)	86.6	±	16.6	78.8	±	15.4	0.089	0.48
MAP (mmHg)	106.2	±	19.9	94.3	±	18.5	0.082	0.62
SVR (dyn•s^−1^•cm^−5^)	1,447.5	±	531.1	1700.0	±	494.4	0.086	0.49
O_2_ pulse (ml•beat^−1^)	4.37	±	2.74	4.08	±	2.78	0.773	0.10
HR (beat•min^−1^)	77.0	±	14.2	83.0	±	13.1	0.290	0.43

values are mean ± standard deviation.

Abbreviations: DS (Down syndrome); VE (minute ventilation); RER (respiratory exchange ratio); RR (respiratory rate); V_T_: tidal volume; VE_q_O_2_: ventilatory equivalent for O_2_; VE_q_CO_2_: ventilatory equivalent for CO_2_; PETO_2_ (end-tidal partial pressure for oxygen); VO_2_ (oxygen uptake); VCO_2_ (carbon dioxide production); Q (cardiac output); SBP (systolic blood pressure); DBP (diastolic blood pressure); MAP (mean arterial pressure); SVR (systemic vascular resistance); HR (heart rate).

*p-value*: between-group differences at rest.

### 3.1 Peak values

Compared to the peak values obtained by the non-DS group, individuals with DS had lower values for ventilation (VE; *p* < 0.001), respiratory exchange ratio (RER; *p* = 0.043), tidal volume (V_T_; *p* = < 0.001), ventilatory equivalent for O_2_ (VEqO_2_; *p* < 0.001), end-tidal partial pressure for O_2_ (P_ET_O_2_; *p* = 0.001), VO_2_ (*p* < 0.001); CO_2_ production (VCO_2_; *p* < 0.001), achieved a lower percentage of the predicted VO_2_ max (*p* < 0.001). The DS group had a higher percentage of the respiratory reserve at the end of the tests (*p* < 0.001). Finally, the test duration of the DS participants was shorter than the test duration of the non-DS group (*p* < 0.001) (Table 3). Regarding the hemodynamic parameters, the DS group had lower peak values for Q (*p* = 0.002), SV (*p* = 0.030), SBP (*p* < 0.001), DBP (*p* = 0.016), mean arterial BP (MAP; *p* = 0.001), O_2_ pulse (*p* < 0.001), and HR (*p* < 0.001) than the non-DS group (Table 3).

**TABLE 3:** Participants’ peak cardiorespiratory and hemodynamic values.

	Non-DS (n = 15)			DS (n = 15)			*p*-value	Effect size
	Peak values			Peak values				
**Cardiorespiratory data**								
VE (L•min^−1^)	123.9	±	23.3	53.7	±	14.5	<0.001	3.61
RER	1.14	±	0.03	1.12	±	0.03	0.043	0.77
RR (breath•min^−1^)	46.3	±	7.4	42.0	±	7.8	0.139	0.56
V_T_ (L)	2.47	±	0.59	1.15	±	0.22	<0.001	2.93
VEqO_2_	33.9	±	3.8	28.2	±	2.6	<0.001	1.75
VEqCO_2_	27.8	±	2.8	27.8	±	2.3	0.992	0.00
P_ET_O_2_ (mmHg)	112.4	±	3.8	107.5	±	3.0	0.001	1.42
VO_2_ (ml•kg^−1^•min^−1^)	51.6	±	10.9	28.8	±	6.7	<0.001	2.53
VO_2_ (L•min^−1^)	3.73	±	0.91	1.90	±	0.44	<0.001	2.55
VCO_2_ (L•min^−1^)	4.26	±	0.98	2.12	±	0.54	<0.001	3.12
VO_2_ max predicted (%)	123.1	±	16.3	77.9	±	10.7	<0.001	3.27
Respiratory reserve (%)	14.3	±	9.6	55.5	±	8.2	<0.001	4.50
Treadmill test duration (min)	16.6	±	2.0	12.5	±	2.1	<0.001	1.99
**Hemodynamic data**								
Q (L•min^−1^)	20.2	±	3.0	14.1	±	3.6	0.002	1.32
Stroke Volume (ml)	113.2	±	20.4	94.2	±	22.9	0.030	0.87
SBP (mmHg)	188.4	±	34.0	141.6	±	22.7	<0.001	1.56
DBP (mmHg)	106.0	±	21.5	85.6	±	17.2	0.016	0.97
MAP (mmHg)	141.6	±	26.8	107.5	±	22.0	0.001	1.36
SVR (dyn•s^−1^•cm^−5^)	794.4	±	315.2	916.3	±	463.8	0.431	0.30
O_2_ pulse (ml•beat^−1^)	20.4	±	4.2	12.5	±	2.5	<0.001	1.96
HR (beat•min^−1^)	183.0	±	7.1	150.0	±	13.1	<0.001	3.04

Note: values are mean ± standard deviation.

Abbreviations: DS (Down syndrome); VE (minute ventilation); RER (respiratory exchange ratio); RR (respiratory rate); V_T_: tidal volume; VEqO_2_: ventilatory equivalent for O_2_; VEqCO_2_: ventilatory equivalent for CO_2_; P_ET_O_2_ (end-tidal partial pressure for oxygen); VO_2_ (oxygen uptake); VCO_2_ (carbon dioxide production); Q (cardiac output); SBP (systolic blood pressure); DBP (diastolic blood pressure); MAP (mean arterial pressure); SVR (systemic vascular resistance); HR (heart rate).

*p-value*: between-group differences for peak values.

### 3.2 Submaximal values

**FIGURE 1:**
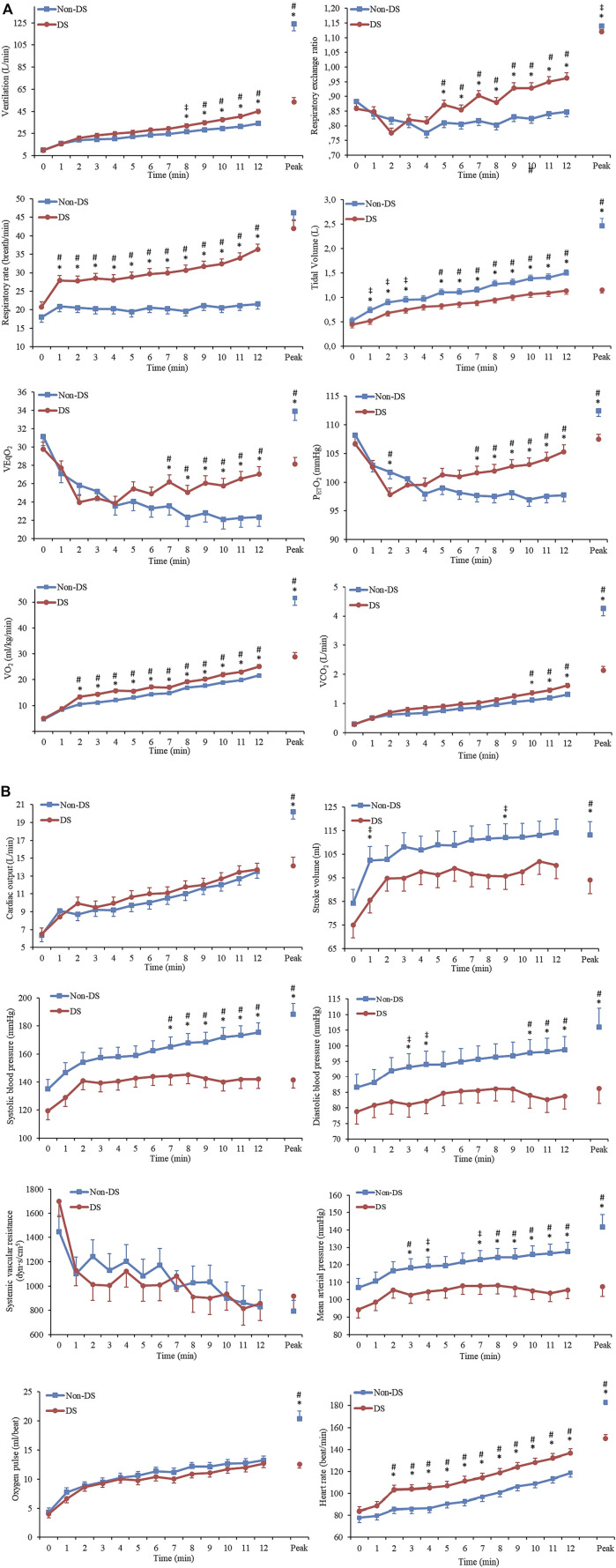
Cardiorespiratory and hemodynamic parameters in response to submaximal exercise in adults with and without Down syndrome. **(A)**: Cardiorespiratory parameters; **(B)**: Hemodynamic parameters. Abbreviations: DS (Down syndrome). Values are mean ± standard error. * Between-group differences (*p* < 0.050). ^‡^ Medium effect size. ^#^ Large effect size.

When analysing the ventilatory submaximal values (Figure 1A), significant interaction effects were observed for all variables (*p* < 0.050). In addition, significant group and time differences were observed for VE; RER; respiratory rate (RR); VEqO_2_; P_ET_O_2_; VO_2,_ and V_T_ (all *p* < 0.050). However, for VCO_2_ and VEqCO_2_, we only found a significant difference in time (*p* < 0.001).

The analysis of the hemodynamic variables (Figure 1B) revealed significant interaction effects and group and time differences for SBP, MAP, and HR (all *p* < 0.010). Even though no significant interactions were observed for DBP, significant group and time differences were observed (all *p* < 0.050). We only found significant time differences for Q, SV, systemic vascular resistance (SVR), and O_2_ pulse (all *p* < 0.010).

**FIGURE 2:**
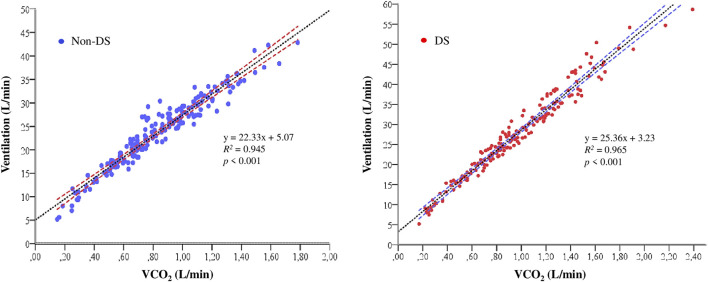
Relationship between ventilation (VE; L/min) and carbon dioxide production (VCO_2_; L/min) during the submaximal phases of the treadmill test. Abbreviations: DS (Down syndrome).

During the submaximal exercise, we found significant correlations between VE and VCO_2_ for both groups (all *p* < 0.001) (Figure 2).

## 4 Discussion

In the present study we observed differences in the cardiorespiratory response between the two groups (DS vs non-DS), both at submaximal and maximal intensities.

At submaximal effort, and comparing the same workloads performed by each group, we found that the response of cardiorespiratory parameters (VE; RER; RR; VO_2_; HR) was higher in the DS group; while some hemodynamic parameters (SBP; DBP; MAP) were attenuated, or similar (Q; SV; SVR; O_2_ pulse) compared with the non-DS group. A better understanding of specific characteristics and physiological demands that submaximal exercise represents for persons with DS, will help researchers, sports scientists, and physical therapists to better design exercise and rehabilitation programs by using adequate submaximal workloads, which are more representative of the metabolic cost and the intensities at which we moved during the activities of daily living.

As far as we know, this is the first time that a study examines and compares cardiorespiratory and hemodynamic parameters in response to maximal and submaximal exercise intensities in age-and sex-matched adults with and without DS. The results attained in our study during the peak exercise support and further substantiate previous studies showing that persons with DS have different physiologic and hemodynamic responses to maximal effort than peers without DS (Fernhall et al., 1996; Baynard et al., 2008; Fernhall et al., 2009; Fernhall et al., 2013).

### 4.1 Peak cardiorespiratory and hemodynamic responses

Concerning the peak cardiorespiratory values, our results reinforce the findings of previous reports, showing that adults with DS have lower peak cardiorespiratory fitness than non-DS adults (Baynard et al., 2008; Mendonca et al., 2010; Mendonca et al., 2011; Beck et al., 2021). The cause of these differences at peak exercise may include central and peripheral mechanisms (Fernhall et al., 2013). Moreover, as suggested by Fernhall et al. (2013), the autonomic dysfunction observed in DS persons may cause their reduced maximal HR.

In addition to lower peak HR, we also observed lower Q values, which negatively affect the peak VO_2_ and the peak work capacity of individuals with DS. Furthermore, DS persons also present typical characteristics, such as micrognathia, tracheal stenosis, macroglossia (Bull, 2020), and impaired mitochondrial energy metabolism, among others (Valenti et al., 2018), limiting their maximal aerobic capacity. Among our participants, macroglossia was observed in all of them, which might have restricted peak ventilation and adversely affected the peak aerobic capacity.

Our results also allowed us to identify that our participants with DS had significant lower peak V_T_ than those without DS, which added to the fact that both groups present equal values for RR, made the quantities of air mobilized by the DS individuals considerably lower at peak intensities. Thus, negatively affecting O_2_ supply and CO_2_ elimination at peak workloads.

Concerning hemodynamic values, we also found that people with DS reached lower values of SV and Q during peak exercise. Different mechanisms could explain these differences. As indicated by Vis et al. (2012b), one of them is that adults with DS may have a smaller left ventricle compared to non-disabled adults. In addition to this, DS participants may have a lower venous return due to a blunted sympathetic control of blood flow (Hilgenkamp et al., 2018), affecting the preload conditions.

The peak values of the SBP, DBP, and MAP of the DS participants may be affected by a reduced peripheral vasoconstrictor control during the cardiopulmonary exercise test (Fernhall et al., 2013; Hilgenkamp et al., 2019). The effect of a lower MAP during the exercise could negatively affect the venous return, compromising the end-diastolic volume and the SV. If we add to all this that DS persons have lower peak HR (Guerra et al., 2003; Fernhall et al., 2013), the Q will be lower, as we found in our results, thus compromising the correct blood volume distribution and the arrival of O_2_ and nutrients needed to match the metabolic demands of peak workloads.

Cardiorespiratory fitness is a strong independent predictor of life expectancy (Strasser and Burtscher, 2018). Therefore, we believe that further research on these parameters are needed to analyse whether long-term increments in maximal exercise power may affect positively the mortality rate in DS persons.

Finally, we observed that peripheral vascular resistance at maximal loads was similar in both groups, and that the peak O_2_ pulse, which reflects the myocardial O_2_ supply and the cardiac functional reserve, was significantly higher in the non-DS. These data confirm that people with DS have a lower cardiac reserve and a much lower capacity than non-DS to increase it during maximal efforts.

### 4.2 Submaximal cardiorespiratory and hemodynamic responses

When performing an analysis of submaximal loads such as the one presented in our study, it is necessary to take into account the absolute workload used in each phase of the test (the need of energy for metabolic and mechanical work) and the duration of the effort. For this reason, we decided to carry out an innovative approach which allowed us to compare the responses of the parameters studied using the same absolute workloads and the same time of the test.

The data of our study showed a higher VO_2_ at submaximal loads in the participants with DS. We hypothesize that there is a lower biomechanical efficiency of gait patterns in adults with DS; therefore, the participants with DS in our study may have higher needs of VO_2_ when walking. Previous findings support this argument, suggesting that persons with DS have reduced walking economy and higher metabolic cost (Agiovlasitis et al., 2009; Mendonça et al., 2009; Agiovlasitis et al., 2015; Agiovlasitis et al., 2018). Unfortunately, the present protocol does not allow us to determine the causes of group differences in VO_2_ when walking at submaximal intensities and compare them with the previous cited studies.

Our results showed that the individuals with DS had higher RR and lower V_T_, which promoted a higher VE in the DS group. Undoubtedly, this produces a lower ventilatory efficiency, as shown by a higher VEqO_2_ in the DS group. Furthermore, after analyzing the slopes of the relationship between VE and VCO_2_ (DS = 25.36 points vs Non-DS = 22.33 points) (Figure 2), we may conclude that participants with DS have a lower ventilatory efficiency, which increases the energy expenditure required by the respiratory muscles for a given workload. On the other hand, the VE-to-VCO2 slopes for both groups are within the normal values, which range from 21 to 31 units (Sun et al., 2002; Naeije and Faoro, 2018), which indicates that the ventilatory response as a function of CO_2_ production during submaximal exercise had a normal behavior in both groups. Together, this indicates that the DS group has a higher ventilatory work and, therefore, higher needs of O_2_ because of a higher respiratory muscle activity, which may also justify the higher VO_2_ at submaximal workloads (Figure 1A).

Moreover, higher RR in the DS group have several consequences, one of which is a higher HR at submaximal workloads compared to control individuals. This fact could explain the increase in HR despite having a lower sympathetic response (Fernhall et al., 2005). These results are consistent with Mendonca et al. (2011) study, where the authors found that DS persons have higher HR than non-DS adults at different submaximal workloads. On the contrary, Vis et al. (2012) found that the HR in DS individuals was lower after performing ten knee bends. Therefore, we hypothesize that in the study of Vis et al. (2012a), there was no compensation increase of the HR because of the short duration of the test (less than 45 s).

The increased ventilatory response could be of central origin and adaptive to the effort secondary to altered gait patterns (Agiovlasitis et al., 2015), neuromuscular abnormalities, and neurological impairments affecting stability and motor control (Zago et al., 2020). The physiological response of DS individuals may be disproportionate to the exercise performed. Moreover, and taking into account that Q is similar in both groups, and VO_2_ is more significant in the DS group, the disproportionate increase in ventilation may be because the peripheral receptors of the carotid body may detect a slight decrease in O_2_ levels, leading to greater ventilation to provide O_2_. By trying to deliver more O_2_ to a more significant number of working muscles during the exercise, the ventilation-perfusion ratio in the DS group would favor ventilation, increasing the P_ET_O_2_ (Figure 1A) in the alveolus and fostering CO_2_ elimination. As shown in Figure 1A, this would also produce an increase in RER, which may not be related to the different metabolic substrates used during the treadmill test but rather a consequence of ventilatory adaptations during submaximal efforts.

As well as during the peak exercise, lower values of SBP, DBP, and MAP were observed in the DS group during submaximal exercise. This could be explained by the autonomic dysfunction affecting persons with DS, which would cause a lower inotropic and chronotropic response. As suggested by previous studies, individuals with DS show signs of reduced peripheral blood flow regulation and vasoconstrictive control during sympathoexcitatory stimulus (Hilgenkamp et al., 2018; Hilgenkamp et al., 2019).

Our study results also show that DS participants, even though they had slightly lower SV values and higher HR, had similar values of Q than the non-DS individuals at submaximal workloads. On the contrary, Vis et al. (2012a) found a reduced SV and Q in adults with DS when performing exercise at submaximal intensity. On the other hand, Pitetti et al. (1992) found that the DS individuals had significantly higher mean Q and similar SV than participants with intellectual disabilities without DS. Such disparity in findings may be due to the different exercise protocols and methodologies implemented to assess hemodynamics parameters.

### 4.3 Limitations

This study presents some limitations. Firstly, we obtained hemodynamic information using a non-invasive finger cuff, which could have been affected by some movement in the left hand in response to changes in the speed or grade of the treadmill. However, we took all necessary precautions to ensure that this did not happen during the tests. Second, we did not assess sedentary or physical activity levels of the participants. These variables could have some effects on the cardiorespiratory and hemodynamic parameters. Thirdly, in our study we included 12 men and three women in each group, which could be considered a limitation due to the effects that sex could have on the parameters studied. Therefore, to prevent that sex may influence the results of the comparisons between groups; we matched the groups by sex. Finally, our findings should be treated with caution due to the sample size and specific age of the participants included in this study, thus having limited generalizability to other age groups. Further research is necessary to corroborate our results in younger and older adults with DS.

## 5 Conclusion

During submaximal exercise, we were able to verify a greater response of HR, VE and VO_2_ for the same absolute workload in the participants with DS. Interestingly, the DS group showed lower, but not significant, SV and similar Q than non-DS participants at submaximal workloads. Moreover, we observed that individuals with DS persons had a reduced blood pressure response to submaximal exercise. On the other hand, the peak values of the parameters analysed in our study reinforce the results obtained in previous studies, demonstrating that people with DS have lower peak values of cardiorespiratory fitness, HR, a lower response of hemodynamic parameters (MAP; SV; Q), and a lower cardiac reserve.

Further research is needed to determine whether different types of training (i.e., strength training, concurrent training, high-intensity interval training, etc.) may elicit modifications and adaptations in submaximal and maximal cardiorespiratory and haemodynamic responses in people with DS.

## Data Availability

The raw data supporting the conclusion of this article will be made available by the authors, without undue reservation.
